# Heavy Metals Bioaccumulation in Tissues of Spiny-Cheek Crayfish (*Orconectes limosus*) from Lake Gopło: Effect of Age and Sex

**DOI:** 10.1007/s00128-017-2098-2

**Published:** 2017-05-05

**Authors:** Magdalena Stanek, Janusz Dąbrowski, Szymon Różański, Bogdan Janicki, Jacek Długosz

**Affiliations:** 1 Department of Biochemistry and Toxicology, Faculty of Animal Breeding and Biology, UTP University of Science and Technology, Mazowiecka St. 28, 85-084 Bydgoszcz, Poland; 2 Department of Ecology, Faculty of Animal Breeding and Biology, UTP University of Science and Technology, Kordeckiego St. 20, 85-225 Bydgoszcz, Poland; 3 Department of Soil Science and Soil Protection, Faculty of Agriculture and Biotechnology, UTP University of Science and Technology, Bernardyńska St. 6, 85-029 Bydgoszcz, Poland

**Keywords:** Heavy metals, Crayfish, Abdominal muscle, Exoskeleton, Sex, Age

## Abstract

The aim of the present work was to assess the concentrations of metals in the abdominal muscle and exoskeleton of 3-year-old males and 4-year-old females and males of spiny-cheek crayfish (*Orconectes limosus*) collected from Lake Gopło. A total of 93 males and 35 females were collected in autumn (October 2014). The analyzes of heavy metals were conducted by means of atomic absorption spectroscopy with a PU9100X spectrometer. The content of mercury was determined using AMA 254 mercury analyser. As analyses indicated heavy metals accumulated in the muscle and exoskeleton in the following sequence: Zn > Cu > Pb > Mn > Ni > Hg and Mn > Pb > Zn > Ni > Cu > Hg, respectively. Statistically significant differences between 3- and 4-year-old males were found for all analyzed metals. Gender dependent differences were calculated only for Ni in the muscle tissue and for Mn and Hg in the exoskeleton. In comparison with the study carried out 2 years ago notably higher concentrations of Pb were found in the muscle and a higher content of Zn, Pb, Mn and Ni was determined in the exoskeleton.

Crayfish may accumulate a high quantity of heavy metals in their tissues whether essential or not and may live in benthic habitats affected by local contamination. It makes crayfish a good bioindicator of environmental pollution (Sánchez López et al. 2004; Rainbow 2007; Kouba et al. 2010; Kuklina et al. 2014; Goretti et al. 2016). Multiple studies have demonstrated that the levels of heavy metals in crayfish depends on factors such as species, location, diet, sex, size and type of tissue (Canli and Furness 1993, Turoczy et al. 2001; Thawley et al. 2004; Naghshbandi et al. 2007). Gender can also be an important factor affecting the level of heavy metals in crustaceans’ bodies (Chen et al. 2005, Yɪlamz and Yɪlmaz 2007; Tunca et al. 2013b; Dincer and Aydin 2014). Zn, Cu, Ni and Mn constitute essential heavy metals in crayfish, having many biological roles and being necessary for the proper functioning of the organism and toxic only in the increased quantities. Non-essential metals as Pb and Hg do not play any role in metabolism and are toxic even at low concentrations (Mackevičienė 2002, Kouba et al. 2010; Protasowicki et al. 2013).

The aim of the present work was to assess concentrations of trace metals Zn, Cu, Mn, Pb, Ni and Hg in the abdominal muscle and exoskeleton of spiny-cheek crayfish (*Orconectes limosus* Rafinesque, 1817) collected from Lake Gopło (Poland) and the relationship between the heavy metal concentrations with sex and age was investigated. Additionally, the mean content of heavy metals of 3- and 4-year-old males was compared with the results obtained from previous research carried out on males crayfish of comparable size (Stanek et al. 2014) (Fig. 1).

**Fig. 1:**
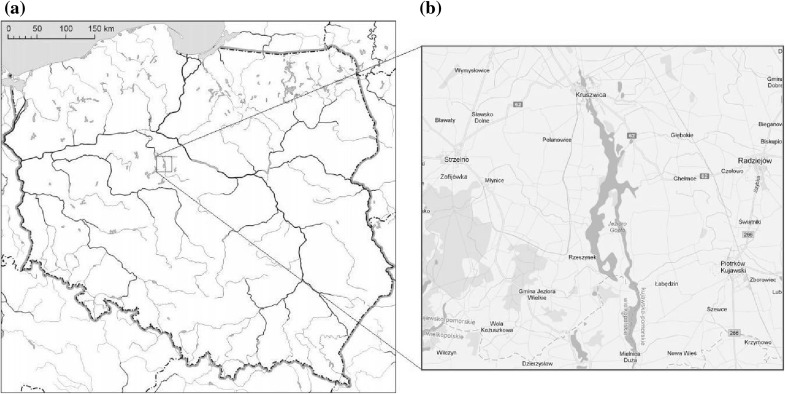
Map of Poland (**a**) and location of Lake Gopło (**b**)

## Materials and Methods

Lake Gopło is a flow-through water body which is located in the southern part of Kuyavian-Pomeranian Province (Fig. 1) with two potential sources of pollution of the lake. The first one is related to agricultural pollutions and the second source usually refers to industrial activities (sugar factory and vegetable fats factory located in Kruszwica) (Szatten 2007; Juśkiewicz et al. 2015).

In Poland is valid the five-stage classification of surface water (five water purity classes: I, II, III, IV and V—with the decreasing purity). The status of surface Polish waters incorporates the ecological status (which includes biological elements, physicochemical and hydromorphological elements) and the chemical status. Studies conducted by Regional Inspectorate for Environmental Protection in Bydgoszcz (WIOŚ from Polish) in years 2008–2013 and in 2015 showed a slow improvement in water quality of the tributaries of Lake Gopło (Report WIOŚ 2014 and 2016). According to these reports, the physico-chemical parameters are good (lake water can be classified as Class I), but the ecological potential is bad and the biological parameters are bad (Class IV—bad purity) what determine a lower grade of water quality. Crayfish were collected in the northern part of the lake which is strongly affected by industrial pollution.

The spiny-cheek crayfish is not native to Europe and its introduction in Poland has resulted in high abundances throughout most of the country (Holdich and Black 2007) and are commonly dominant because invaded almost the entire area of Polish waters, with the exception of the south-east part (Krzywosz 2004). This species of crayfish has a small chelipeds with little meat, but it may be a consumer object for local people, like noble crayfish (*Astacus astacus* Linnaeus, 1758), although it is not exploited on a scale as fish caught from this reservoir. This crayfish is immune to the crayfish plague, because it carries a novel genotype of crayfish plague pathogen Aphanomyces astaci, so it’s a threat to native noble and mud crayfish (*Astacus leptodactylus* Esch.) species. Spiny-cheek crayfish is tolerant of a wide range of environmental conditions including locations that have been affected by chemical pollution and eutrophication. A detailed description of morphological parameters, age of sexual maturity, the maximum achievable size, weight and total length and life expectancy of this species of crayfish are presented by Stanek et al. (2014) based on previous data published by Mastyński (1999).

A total of 93 males and 35 females of the spiny-cheek crayfish were caught in autumn (October 2014) after a period of intense feeding using fyke nets. Traps were placed in the litoral zone of the lake at a depth of 1–5 m. Crayfish with damaged claws were not taken into account for further analyses. There were sexually mature individuals of total length from 8.2 to 11.0 cm (measured from the tip of the rostrum to the end of the telson) (Table 1). Spiny-cheek crayfish become sexually mature in Polish climatic conditions at the end of the second growing season (Holdich and Black 2007), when they reach a body length of 6 cm (Pielplow 1938; Juchno and Chybowski 2003). Spiny-cheek crayfish in Poland mate in the autumn but females do not lay their eggs until spring (the end of April and beginning of May). The study included males with the well-developed the first pair of pleopods form I of males, according to description of Chybowski (2007). Females were in the same stage of reproductive cycle. Due to the relatively low amounts of the meat obtained from the abdomen part of individual crayfish, the muscles from two individuals with the same body length were combined. For analysis, the abdominal muscle and exoskeleton were dissected. Exoskeleton was collected before it’s molting by crayfish, when was hard and the levels of heavy metals could be maximal. Exoskeleton was air-dried and the muscle samples were freeze dried in Lyovac GT2 freeze-drier by Finn-Aqua (Finland).

**Table 1:** Biometric measurements of the analysed crayfish spiny-cheek (*Orconectes limosus*) caught from Lake Gopło

Individuals		n	Total length (cm) min–max (mean)	Body mass (g) min–max (mean)
3-Year-old	Males	61	8.2–9.5 (8.9)	19.5–30.0 (25.6)
4-Year-old	Females	35	9.6–11.0 (10.3)	27.1–46.4 (34.4)
	Males	32	9.7–11.0 (10.1)	28.2–44.4 (23.0)

Heavy metal concentrations were determined in freeze dried samples after *aqua regia* digestion (ISO 11466) using atomic absorption spectroscopy (AAS) with a PU9100X spectrometer. Total mercury content was determined in solid samples after thermal decomposition in 700 °C on single-purpose atomic absorption spectrometer AMA-254 (Altec, Czech Republic). Certified AAS Merck standard solutions were used for the calibration of the standard curves, and validation was conducted on Certified Reference Material Fish Muscle ERM^®^-BB422 and Certified Reference Material Sandy Loam Soil CRM027-050 (Table 2). The concentrations of the metals were calculated from linear calibration plots obtained by measurement of the standard solutions. All determinations were made in triplicate and the data for samples of muscle were corrected to oven-dry (105 °C) moisture content. Concentrations of heavy metals in spiny-cheek crayfish tissues were expressed as mg kg^−1^ dry weight.

**Table 2:** Total content of elements in certified materials

Element	Certified value (mg kg^−1^)	Determined value (mg kg^−1^)	SD (%)
Zn	16.0 ± 1.1^a^	15.2 ± 1.4	1.26
Cu	1.67 ± 0.16^a^	1.82 ± 0.22	0.16
Pb	51.9 ± 2.46^b^	47.26 ± 3.23	2.23
Mn	0.368 ± 0.028^a^	0.388 ± 0.033	0.02
Ni	10.5 ± 0.704^b^	8.91 ± 0.87	0.32
Hg	0.601 ± 0.030^a^	0.583 ± 0.021	0.03

^a^Certified Reference Material ERM^®^-BB422

^b^Certified Reference Material CRM027-050

Data analyses were performed with using Statistica 8.0 software (StatSoft, USA). The normality of all data was tested using the Shapiro–Wilk’s test, and the homogeneity of variance was tested with Levene’s test. Significance of differences in the average content of metals in muscle and exoskeleton (tissues dependent differences), differences between 3-year-old and 4-year-old males (age dependent differences) and differences between 4-year-old females and males (gender dependent differences) were calculated by T-test. The division of crayfish on the age groups was made on the basis of their body length, in accordance with Pielplow (1938). In order to eliminate the interaction between the independent variables (age and the total length), one-way analysis of covariance (ANCOVA) was used for testing age dependent differences in the concentration of heavy metals with the total length of crayfish as the covariate. They were met assumptions for ANCOVA about the linear relationship between dependent variables and the covariate and homogeneity regression slopes. Those analyses confirmed statistically significant differences in concentrations of all heavy metals between 3- and 4-year-old males.

## Results and Discussion

Our analyses indicated that heavy metals accumulated in the following sequence: Zn > Cu > Pb > Mn > Ni > Hg in the muscle and Mn > Pb > Zn > Ni > Cu > Hg in the exoskeleton and there were statistically significant differences in all heavy metals between analyzed tissues (*p* = 0.000) (Table 3).

**Table 3:** Heavy metals concentrations in the abdominal muscle and exoskeleton of spiny-cheek crayfish (*Orconectes limosus* Raf.) caught from Lake Gopło

Heavy metals (mg kg^−1^)	Abdominal muscle	Exoskeleton
Zn	67.39 ± 8.77^a^	15.97 ± 2.91^b^
Cu	19.89 ± 4.68^a^	1.48 ± 0.69^b^
Pb	14.94 ± 3.36^a^	19.97 ± 2.48^b^
Mn	14.19 ± 2.22^a^	145.10 ± 46.95^b^
Ni	10.56 ± 2.01^a^	15.73 ± 0.95^b^
Hg	0.27 ± 0.03^a^	0.02 ± 0.02^b^

The values in the same row marked with different letters (a, b) differ statistically significant at *p* ≤ 0.05 (T-test)

Very similar results denoted Mackevičienė (2002), Naghshbandi et al. (2007), Kouba et al. (2010) and Goretti et al. (2016) in noble, mud and red swamp crayfish (*Procambarus clarkii*, Girard), respectively. High Zn and Cu contents in crayfish confirmed Alcorlo et al. (2006), who showed that the content of these metals in the meat does not depend of their quantities in the environment.

**Fig. 2:**
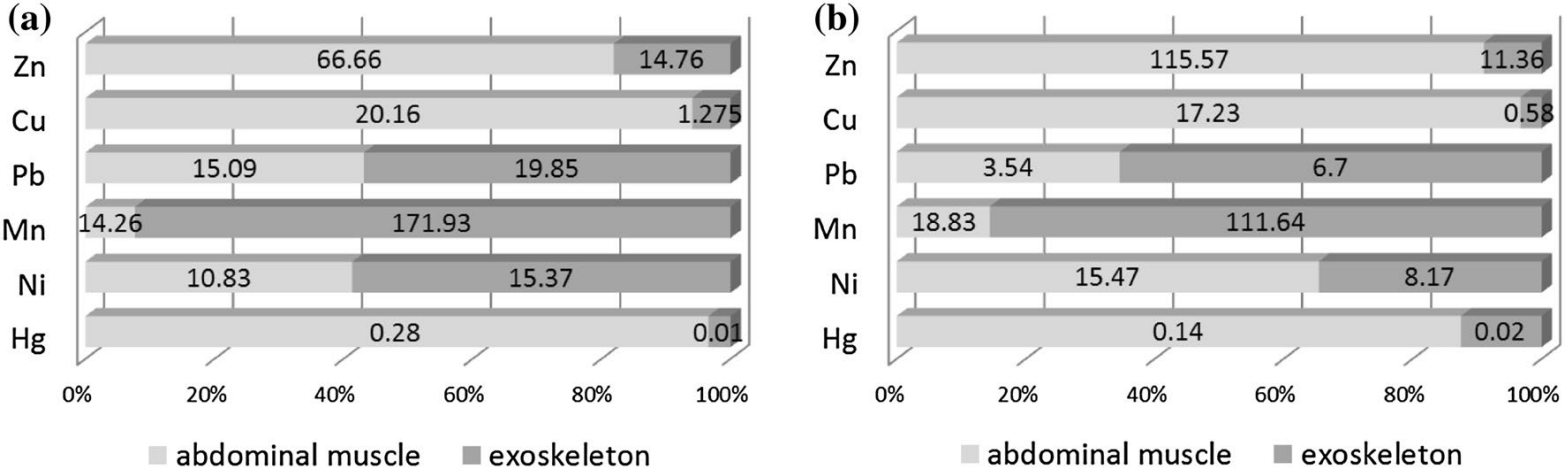
Metal content ratios (abdominal muscle/exoskeleton) in 3- and 4-year-old males caught in 2014 (**a**) and in males from 2012 (**b**) (Stanek et al. 2014)

Zn accumulated in a higher amounts in the muscle than in exoskeleton (Table 3). Naghshbandi et al. (2007) and Protasowicki et al. (2013) determined the same results in comparison with Mackevičienė (2002) who observed a higher concentrations of Zn in exoskeleton that might indicate that this tissue is involved in the excretion of this metal from the body. Numerous studies confirm that exoskeleton growth and moulting may be responsible for metals excretion from organism (Weeks et al. 1992; Bergey and Weis 2007) but mechanism of detoxification may vary depending on the particular element and crustacean species (Keteles and Fleger 2001). Moreover, in the present work were statistically significantly differences in Zn content between individuals of different ages in the muscle (*p* = 0.000) (Table 4) as opposed to exoskeleton (*p* = 0.241) (Table 5). These results were confirmed by Suárez-Serrano et al. (2010) although other studies have shown that muscle tissue contains the lowest heavy metal levels compared with other tissues (Alcorlo et al. 2006). Previous studies concerning metal contents in crayfish caught in spring from Lake Gopło demonstrated much higher level of Zn in the muscle (115.57 mg kg^−1^) and lower in exoskeleton (11.36 mg kg^−1^) (Stanek et al. 2014) (Fig. 2). Figure 2 shows proportions in average content of metals in abdominal muscle and meat of crayfish collected in 2014 compared with data from 2012.

**Table 4:** Heavy metals concentrations in the abdominal muscle of 3-year-old males and 4-year-old females and males of spiny-cheek crayfish (*Orconectes limosus*) caught from Lake Gopło

	Zn	Cu	Pb	Mn	Ni	Hg
3-Year-old ♂	58.18^a,A^ (±3.19)	15.60^a,A^ (±2.09)	12.31^a,A^ (±3.08)	12.91^a,A^ (±1.49)	8.82^a,A^ (±0.86)	0.25^a,A^ (±0.01)
4-Year-old ♂	75.14^b,x,A^ (±2.15)	24.72^b,x,A^ (±1.37)	17.87^b,x,A^ (±2.90)	15.60^b,x,A^ (±1.21)	12.84^b,x,A^ (±0.47)	0.31^b,x,A^ (±0.01)
4-Year-old ♀	70.67^x^ (±7.45)	20.22^x^ (±4.24)	15.18^x^ (±1.11)	14.30^x^ (±3.05)	10.36^y^ (±1.72)	0.27^x^ (±0.03)

The values in the same column marked with different letters differ statistically significant at *p* ≤ 0.05

a,b—Between 3-year-old and 4-year-old males (age dependent differences) (T-test)

x,y—Between 4-year-old females and males (gender dependent differences) (T-test)

A,B—One-way analysis of covariance (ANCOVA) with the total length as a covariate

**Table 5:** Heavy metals concentrations in the exoskeleton of 3-year-old males and 4-year-old females and males of spiny-cheek crayfish (*Orconectes limosus*) caught from Lake Gopło

	Zn	Cu	Pb	Mn	Ni	Hg
3-Year-old ♂	13.28^a^ (±1.89)	1.17^a^ (±0.46)	20.10^a^ (±1.34)	192.24^a^ (±26.76)	15.25^a^ (±0.33)	0.01^a^ (±0.00)
4-Year-old ♂	16.24^a,x^ (±3.23)	1.38^a,x^ (±0.90)	19.60^a,x^ (±4.71)	151.62^a,x^ (±9.24)	15.49^a,x^ (±1.40)	0.01^a,x^ (±0.00)
4-Year-old ♀	18.38^x^ (±0.48)	1.89^x^ (±0.70)	20.21^x^ (±0.51)	91.44^y^ (±17.29)	16.45^x^ (±0.56)	0.05^y^ (±0.02)

The values in the same column marked with different letters differ statistically significant at *p* ≤ 0.05 (t-test)

a,b—Between 3-year-old and 4-year-old males (age dependent differences)

x,y—Between 4-year-old females and males (gender dependent differences)

Our analyses indicated that Cu was accumulated in the greatest amounts in the muscle than in the exoskeleton (Table 3). The opposite results were denoted by Mackevičienė (2002) for noble crayfish. There were statistically significant differences in Cu content between individuals of different ages only in the muscle (*p* = 0.000) (Table 4). As our previous analysis showed, mean content of Cu was very similar in the muscle (17.23 mg kg^−1^) and lower in the exoskeleton (0.58 mg kg^−1^) (Stanek et al. 2014) in comparison with the presented data (Fig. 2).

Concentrations of Pb were significantly higher in exoskeleton tissues of spiny-cheek crayfish in comparison to muscle (*p* = 0.001) (Table 3). The similar results were observed by Madigosky et al. (1991), Mackevičienė (2002) and Alcorlo et al. (2006). Because Pb is neither essential nor beneficial to living organisms (Kouba et al. 2010), it seems that the accumulation of this metal in exoskeleton in a large amounts might indicate that this tissue is involved in the excretion of this metal. Pb is detoxified by metallothioneins or phosphoric granules stored permanently in tissues (Mackevičienė 2002, Alcorlo et al. 2006; Bergey and Weis 2007). Our analyses indicated there were statistically significant differences in Pb content in the muscle between males of different ages (*p* = 0.013) (Table 4) as opposed to exoskeleton (*p* = 0.867) (Table 5). It’s worth noting the much higher content of this metal in the muscle, when compared to data from 2014 (3.54 mg kg^−1^) (Stanek et al. 2014) (Fig. 2). This could be probably caused by the activities of open-pit brown-coal mine near Kruszwica (in Tomisławice) in operation since the Stanek et al. (2014) study. But this hypothesis requires further investigations.

Mn accumulation in high concentrations may have a toxic effect (Tunca et al. 2013b). Our analysis indicated statistically significant differences in Mn content between the muscle and exoskeleton (*p* = 0.000) (Table 3). These values were very similar to those obtained in 2012 (Stanek et al. 2014) for the muscle (18.83 mg kg^−1^) and for the exoskeleton (111.64 mg kg^−1^) (Fig. 2). The high Mn concentration in the exoskeleton (in comparison to muscle) might indicate that this tissue is involved in the excretion of this metal (Mackevičienė 2002). Statistically significant differences were observed in the content of Mn between 3- and 4-year-old spiny-cheek males in the muscle (*p* = 0.010) (Table 4).

Ni is considered to be essential to various biological functions in low amounts (Kouba et al. 2010), but it is still unclear whether this metal is essential for crayfish (Tunca et al. 2013a). Our analysis indicated significantly higher concentration of Ni in spiny-cheek were determined in the exoskeleton in comparison with the muscle (*p* = 0.000) (Table 3). In addition, there were significant differences determined for muscle Mn concentrations between individuals of different ages (*p* = 0.000) (Table 4). These differences were not observed in the exoskeleton (*p* = 0.788) (Table 5). Mackevičienė (2002) and Protasowicki et al. (2013) reported a higher levels of Ni in the exoskeleton. This is contrary to the results of Stanek et al. (2014) where higher Ni concentrations were recorded in muscle (15.47 mg kg^−1^) relative to exoskeleton (8.67 mg kg^−1^) (Fig. 2).

Our analysis indicated a highly statistically significant differences in Hg content between the muscle and exoskeleton (*p* = 0.000) (Table 3) and these values were very similar to those determined in the previous study which were 0.14 mg kg^−1^ in the muscle and 0.02 mg kg^−1^ in the exoskeleton (Stanek et al. 2014) (Fig. 2). Similar results were observed by Protasowicki et al. (2013). Moreover, there were significant differences between 3-year-old and 4-year-old males in the muscle tissue (*p* = 0.000) (Table 4), that were similar to the results of Chouvelon et al. (2009) and Elahi et al. (2012). EU directive (EU regulation, 2011) indicates that the maximum levels of Hg in the muscle of crayfish should not exceed 0.5 mg kg^−1^ wet weight. Food and Drug Administration (FDA) and U.S. Environmental Protection Agency (EPA) have both established action limits 1.0 and 0.5 mg Hg kg^−1^ respectively, for mercury concentration in fish and crayfish (Schuler et al. 2000). Suárez-Serrano et al. (2010) determined high Hg concentrations (3.5 mg kg^−1^) in the muscle of crayfish captured near the sediment waste.

Differences in the heavy metals content between individuals of different sexes were analyzed, but statistically significant differences were detected only for Ni in the muscle (*p* = 0.014) (Table 4) and for Mn (*p* = 0.006) and Hg (*p* = 0.008) in the exoskeleton (Table 5). The highest gender differences were recorded for Mn in the exoskeleton. The concentration of this metal was lower in females than males what may result from the fact that Mn plays an important role in the gametogenesis of crustaceans and large amount of this metal may be accumulated in the ovary. Analyzed females were in the same stage of reproductive cycle. It could explain the reduced amount of Mn in the exoskeleton of females in comparison with males. Kurun et al. (2010), Protasowicki et al. (2013) and Tunca et al. (2013b) confirmed lower amounts of Mn in the muscle of females than males. Our analyses of spiny-cheek crayfish indicated a higher concentration of Hg was detected in the muscle (Table 4) but gender dependent differences were only observed for exoskeleton tissues (Table 5). Elahi et al. (2012) indicated that Hg concentrations in the muscle were significantly higher in female of prawns (*Penaeus semisulcatus* De Haan, 1844) (0.22 mg kg^−1^) than males (0.15 mg kg^−1^) and gender dependent differences in Hg content may be due to differences in diet and habitat. There were no statistically significant differences between sexes for other metals as opposite to Canli and Furness (1993) who determined that levels of several metals in various tissues differed between the sexes of the Norway Lobster (*Nephrops norvegicus*, Linnaeus, 1758) and to Naghshbandi et al. (2007) who confirmed differences in Zn and Cu content in the muscle between sexes for mud crayfish. As Chen et al. (2005) confirmed, Cu is an essential metal for the blood pigment in crustaceans and therefore there shouldn’t be no differences between sexes. Similary, Yɪlamz and Yɪlmaz (2007) and Dincer and Aydin (2014) reported that sex wasn’t an important factor influencing the content of Ni.

1. Higher concentrations of essential elements (Zn, Cu) were found in the muscle tissue in contrast to exoskeleton, where a higher content of non-essential metal (Pb) was determined. It may indicate that exoskeleton is involved in the excretion of toxic metals from the body of this species.
2. Metal concentrations in the muscle of spiny-cheek crayfish significantly depended on age (two size classes which differed statistically significantly) in the cases of all analyzed metals. It confirms that the period of the exposure to the environmental factor plays a significant role in the level of the metals accumulation.
3. No sex related differences were evident with respect to metal bioaccumulation in either muscle or exoskeleton tissues.
4. Content of the toxic metals in the muscle of spiny-cheek from Lake Gopło didn’t exceed the statutory limits for fish and crayfish intended for human consumption (except Pb). Because contamination of crayfish by heavy metals may pose a real risk to consumer, therefore, it is important to have knowledge on heavy metals levels in the tissues of crayfish used for food, and further investigation should be continued in future studies.
